# Human LINE-1 retrotransposon induces DNA damage and apoptosis in cancer cells

**DOI:** 10.1186/1475-2867-6-13

**Published:** 2006-05-02

**Authors:** S Mehdi Belgnaoui, Roger G Gosden, O John Semmes, Abdelali Haoudi

**Affiliations:** 1 Department of Microbiology and Molecular Cell Biology, Eastern Virginia Medical School, Norfolk, Virginia 23507, USA; 2 Center for Reproductive Medicine and Infertility, Weill Medical College, Cornell University, New York, NY 10021, USA

## Abstract

**Background:**

Long interspersed nuclear elements (LINEs), Alu and endogenous retroviruses (ERVs) make up some 45% of human DNA. LINE-1 also called L1, is the most common family of non-LTR retrotransposons in the human genome and comprises about 17% of the genome. L1 elements require the integration into chromosomal target sites using L1-encoded endonuclease which creates staggering DNA breaks allowing the newly transposed L1 copies to integrate into the genome. L1 expression and retrotransposition in cancer cells might cause transcriptional deregulation, insertional mutations, DNA breaks, and an increased frequency of recombinations, contributing to genome instability. There is however little evidence on the mechanism of L1-induced genetic instability and its impact on cancer cell growth and proliferation.

**Results:**

We report that L1 has genome-destabilizing effects indicated by an accumulation of γ-H2AX foci, an early response to DNA strand breaks, in association with an abnormal cell cycle progression through a G2/M accumulation and an induction of apoptosis in breast cancer cells. In addition, we found that adjuvant L1 activation may lead to supra-additive killing when combined with radiation by enhancing the radiation lethality through induction of apoptosis that we have detected through Bax activation.

**Conclusion:**

L1 retrotransposition is sensed as a DNA damaging event through the creation DNA breaks involving L1-encoded endonuclease. The apparent synergistic interaction between L1 activation and radiation can further be utilized for targeted induction of cancer cell death. Thus, the role of retrotransoposons in general, and of L1 in particular, in DNA damage and repair assumes larger significance both for the understanding of mutagenicity and, potentially, for the control of cell proliferation and apoptosis.

## Background

Retrotransposons are mobile retroelements that utilize reverse transcriptase and RNA intermediates to relocate within the cellular genome. Retrotransposons are subdivided into two subclasses: LTR-(long terminal repeats) and non-LTR-retrotransposons. LINE-1 (Long Interspersed Nuclear Element type 1, or L1), is the most common family of non-LTR retrotransposons in the human genome; with about 500,000 copies, it comprises about 17% of the genome [1,2]. Only a fraction of L1 elements in the human genome are intact: most are truncated (usually at the 5' -end) and mutated (often at multiple sites). However, there are still about 80–100 retrotransposition-competent L1 elements (RC-L1s) in the genome. Most RC-L1 sequences are evidently silenced by methylation [3] and, possibly, also by the RNA interference pathway [4]. Genomic demethylation after deleting DNA methyltransferase 1 can trigger L1 elements to become mobilized [5].

L1 elements encode proteins necessary for their own mobilization. L1 encodes a 40 kDa (p40) protein (ORF1p) with RNA-binding activity [6], and ORF2p produces a 150 kDa protein with endonuclease [7] and reverse transcriptase [8,9] activities. L1 integrates into the genome by target-primed reverse transcription (TPRT) using the free 3'-OH at the endonuclease cut site on the genomic DNA as a primer and the L1 RNA as a template [10]. ORF1p and ORF2p preferentially associate with their encoding transcript to form a ribonucleoprotein particle (RNP), which is a proposed retrotransposition intermediate.

The retrotransposition of L1 elements requires the integration into chromosomal target sites using L1-encoded endonuclease [7]. L1 endonuclease creates staggering DNA breaks allowing the newly transposed L1 copies to integrate into the genome. Despite the small number of RC-L1s, and the constraints placed upon their movement by cis-preference [11], characterization of retrotransposition events using tagged RC-L1 clones in cultured cells indicate that about 10% of L1 insertions are accompanied by large chromosomal rearrangements, suggesting that active L1s could also lead to genomic instability [12,13].

While the properties of L1-encoded enzymes have been studied extensively in vitro [10], the biological impact of retroelements on normal and cancer cells requires clarification and has been difficult to assess. We propose to test the ability of RC-L1 to induce targeted DNA strand breaks as a mechanism for inducing apoptosis in human cancer cells. Although several reports exist that L1 induces genomic instability, a precise mechanism of action and especially its impact on cell growth is still generally lacking. It is essential that a clear mechanistic model needs to be established to provide a clear understanding of how human L1 retrotransposition is sensed as a DNA damaging event. Here we report that L1 has genome-destabilizing effects indicated by an accumulation of γ-H2AX foci, an early response to DNA strand breaks, in association with induction of apoptosis in breast cancer cells.

## Results

### RC-L1 expression and retrotransposition assay

**Figure 1:**
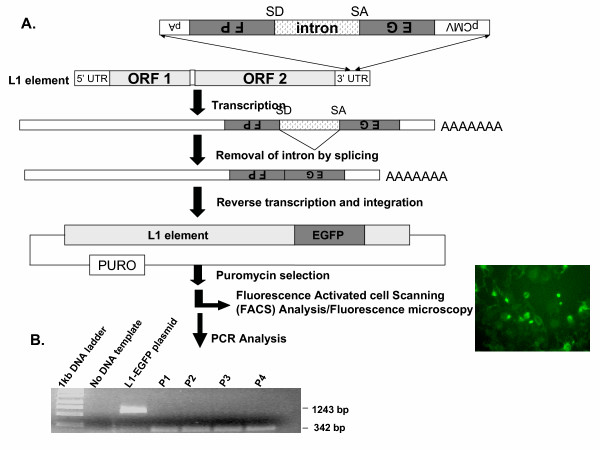
**Experimental strategy for assaying RC-L1 expression and retrotransposition**. (A) L1 element consists of the 5'- and 3'-UTRs and two ORFs. The EGFP retrotransposition cassette is cloned into the L1 3'-UTR in the antisense orientation. L1 elements tagged with the EGFP cassette are cloned into the pCEP4-based mammalian expression vector with puromycin resistance gene. RC-L1-EGFP-expressing cells are sorted by FACS and retrotransposition is detected by EGFP fluorescence using fluorescence microscopy. (B) Confirmation of L1 retrotransposition by PCR as revealed by a 342 bp product. MCF-7 cells-expressing L1-EGFP. Serial passaged cells (P1–P4) show the spliced form of EGFP at 342 bp. No DNA template used as negative control. L1-EGFP plasmid showing the unspliced form at 1243 bp used as a positive control. 1 Kb ladder used as a molecular weight marker.

To monitor EGFP-tagged RC-L1 expression and retrotransposition, we have used a retrotransposition assay that has previously been shown to efficiently detect and monitor L1 retrotransposition events in different cell lines [16-18]. To test whether breast cancer cells can allow RC-L1 expression and retrotransposition, MCF-7 cells were stably transfected with a human RC-L1 tagged with an EGFP antisense cassette, which is interrupted by a γ-globin intron allowing L1 retrotransposition only after splicing of the intron [17] (Fig. 1). The detection of cells expressing EGFP is indicative of delivery, expression and retrotransposition of RC-L1 in these cells (Fig. 1). In order to confirm that resistant clones generated after selection harbor an "active" spliced form of RC-L1, we performed a PCR analysis to verify whether the γ-globin intron has been removed by splicing during L1 retrotransposition events. New retrotransposition events were detected by the presence of the spliced form of EGFP with 342 bp size.

### RC-L1 ORFs are expressed in MCF-7 cells

**Figure 2:**
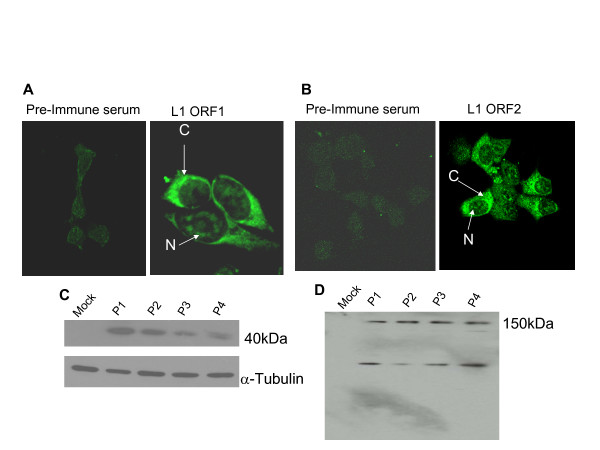
**RC-L1 is expressed in breast cancer cells**. RC-L1- expression determined by immunofluorescence using anti-L1 ORF1 (A) and anti-L1 ORF2 (B) rabbit polyclonal antibodies showed cytoplasmic "C" and nucleolar "N" staining. No specific staining was detected when using pre-immune serums as control. Immunoblotting of whole cell lysates using anti-ORF1 (C) or anti-ORF2 (D) detected a 40 kD and a 150 kDa respectively. Additional bands were detected by anti-ORF2 antibody at 135 kDa and 66 kDa which may be due to cleavage by a cellular protease. P1 through P4 correspond to consecutive passages of RC-L1 expressing cells. Anti-α-tubulin was used as a loading control. Mock transfected cells serve as a negative control.

To check whether EGFP-expressing cells harboring RC-L1 were expressing the L1 proteins necessary for retrotransposition, we investigated the presence of both L1-ORF1 and L1-ORF2 products by immunostaining (Fig. 2a and 2b) and by immunoblotting (Fig. 2c and 2d) using anti-ORF1 and anti-ORF2 rabbit polyclonal antibodies. ORF1 and ORF2 proteins were detected in RC-L1-expressing MCF-7 cells mainly in the cytoplasm and in nucleolar sites consistent with previous reports [9].

### RC-L1 expressing cells exhibit an abnormal cell cycle progression and a DNA damage recognition response

**Figure 3:**
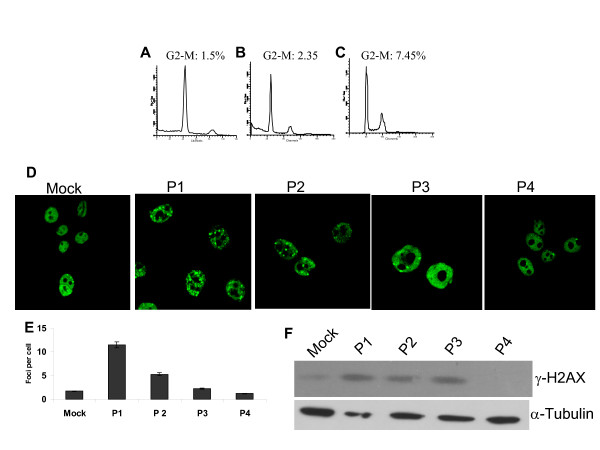
**Cell cycle and DNA damage response analysis of RC-L1-expressing cells**. MCF-7 cells either Mock transfected, expressing GFP or RC-L1 were analyzed by FACS as described under "Experimental Procedures". The histograms represent the distribution of cells through the cell cycle measured by flow cytometry and analyzed with ModFit. (A) untransfected MCF-7 cells. (B) MCF-7 cells transfected with EGFP (C) MCF-7 cells transfected with RC-L1. The percentage of cells in G_2 _or M is shown for each treatment group. Detection of the induction of γ-H2AX foci formation using anti-γ-H2AX polyclonal antibody. (D) by immunostaining of Mock transfected cells and serially passaged RC-L1-expressing cells (P1-P4) (E) Number of γ-H2AX foci in four different cell passages, P1-P4. Error bars show s.d. (F). Expression level of γ-H2AX determined by immunoblotting.

In order to check whether RC-L1 expression and retrotransposition impacts on cell cycle progression, MCF-7 cells were tested for cell cycle profile and DNA damage response following RC-L1 expression and retrotransposition. We analyzed the cell cycle status in the presence of either GFP (control) or RC-L1 in MCF-7 cells. The percentage of cells accumulating in 4N following RC-L1 expression was ~5-fold over non-transfected cells (Fig. 3A–C) while cells transfected with GFP show no significant increase in 4N when compared with non-tranfected cells.

An early response to DSBs is phosphorylation of H2AX, a variant form of the histone H2AX. Phosphorylated H2AX, termed γ-H2AX, can be observed over several megabases flanking the DSB [19]. The role of H2AX and the proteins that accumulate at the site of DSBs is promoting survival of the cells [20]. The presence of γ-H2AX provides the platform for other damage proteins such as 53BP1, Mre11 and Brac1 to localize to the break site [21]. To determine whether RC-L1 expression and retrotransposition is sensed as a DNA damaging event, we checked for the activation and accumulation of γ-H2AX in discrete sites known as DNA damage repair foci. The histone γ-H2AX is rapidly phosphorylated at the sites of DNA double-strand breaks (DSBs) [19]. Interestingly, γ-H2AX is activated and appears as discrete nuclear foci in RC-L1-expressing cells, suggesting that integration had induced such breaks in DNA (Fig. 3D–F).

### Induction of apoptosis in RC-L1-expressing cells

**Figure 4:**
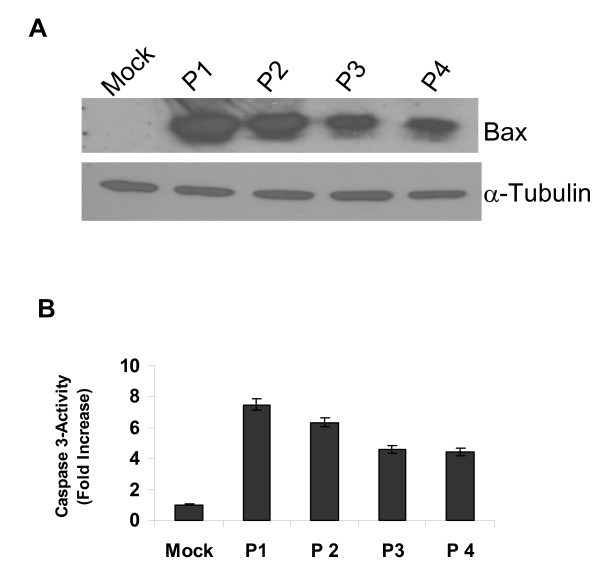
**Induction of apoptosis in breast cancer cells harboring RC-L1**. (A) Pro-apoptotic bax gene expression level determined by immunoblotting using anti-Bax polyclonal antibody. Anti-α-tubulin was used as a loading control. Mock transfected cells serve as a negative control. (B). Induction of apoptosis in RC-L1-expressing cells determined using caspase 3 assay.

Since induction of apoptosis is an early response to DNA damage, we examined to what extent it is induced in MCF-7 cells expressing RC-L1. Expression of a pro-apoptotic gene was analyzed by immunoblotting using anti-Bax antibody. Bax expression was induced in RC-L1-expressing cells passaged for up to 4 consecutives passages (P1-P4), but declined with passage number (Fig. 4A). Apoptosis induction in MCF-7 cells expressing RC-L1 was also tested using a caspase 3 assay. Caspase 3 is an active cell-death protease involved in the execution phase of apoptosis. There was a seven-fold increase in expression of this marker of apoptosis in comparison with mock-transfected cells (Fig. 4B). The level of expression diminished with additional cell passages. These data corresponded closely to the levels of DNA damage observed using the γ-H2AX foci assay, and they demonstrated that induction of apoptosis is associated with the presence of a retrotransposition-competent L1 in MCF-7 cells.

### RC-L1 retrotransposition in p53 mutant cells

**Figure 5:**
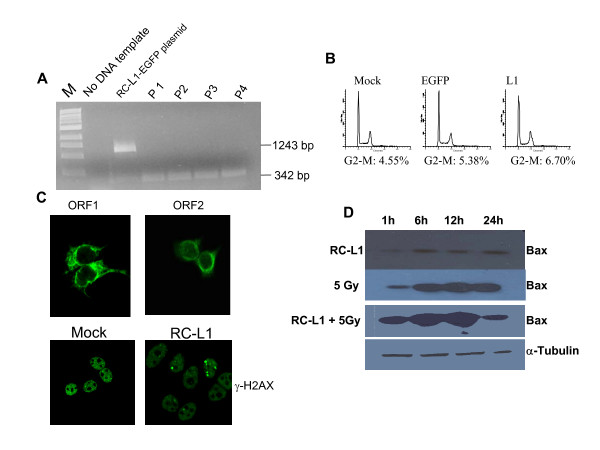
**Impact of RC-L1 expression and retrotransposition on p53 mutant cells**. (A) Confirmation of L1 retrotransposition by PCR as revealed by a 342 bp product. (B) The histograms represent the distribution of cells through the cell cycle measured by flow cytometry and analyzed with ModFit. "A" untransfected T47D cells. "B" T47D cells transfected with EGFP "C" T47D cells transfected with RC-L1. (C) RC-L1- expression determined by immunofluorescence using anti-L1 ORF1 and anti-L1 ORF2 rabbit polyclonal antibodies (upper panels). Detection of the induction of γ-H2AX nuclear foci formation using anti-γ-H2AX polyclonal antibody (lower panels). (D) Pro-apoptotic bax gene expression level determined by immunoblotting using anti-Bax polyclonal antibody in T47D cells either expressing RC-L1, or treated with 5Gy radiation or both and assessed at the indicated time points.

We have previously shown that the impact of L1 expression and retrotransposition on the target cell is dependent upon the p53 status [14]. To further clarify whether p53 mutant cells will sense L1 expression and retrotransposition through the same mechanism as p53 wild type cells, we expressed RC-L1 in T47D breast cancer p53 mutant cells. We first checked for correct splicing of EGFP-L1 construct (figure 5A), then asked whether RC-L1 will have any impact the cell cycle profile. We found that in T47D with mutant p53, RC-L1 does not seem to significantly alter cell cycle progression in these cells (figure 5B). To ensure that RC-L1 is appropriately expressed in these cells, we performed an immunobloting analysis of T47D cells transfected with RC-L1 and probed with anti-L1 ORF1 or anti-L1 ORF2 antibodies. We were indeed able to detect ORF1 and ORF2 proteins mainly in the cytoplasm of these cells with occasionally some staining in the nucleolus, in particular with anti-L1 ORF2 antibody.

We then checked for RC-L1 ability to activate H2AX in these cells. We did not detect any significant activation of H2AX after RC-L1 expression and retrotransposition in comparison with the level of H2AX activation in MCF-7 cells expressing RC-L1 (figure 5, panel C).

Since T47D cells do not seem to allow similar RC-L1 retrotransposition level as MCF-7 cells with wild type p53, we then postulated that simultaneously inducing DSBs via γ-irradiation and L1 activation will possibly swamp the capacity of DNA repair pathways to process Radiation Induced DSBs (RIDSBs). We further postulated that RIDSBs and L1-induced DSBs will competitively sequester γH2AX (and the associated repair protein complexes), resulting in sub-optimal levels of the "repairsome" at each loci; which will delay the processing of the DSBs. In the case of RIDSBs this increased persistence may result in an increased conversion to chromosomal aberrations or may trigger apoptotic cell death. Thus the co-administration of RC-L1 may be a powerful adjuvant to radiation in tumors that are inherently resistant to radiation. Alternatively, the additional sequestration of γ-H2AX/NHEJ complex by RIDSBs may serve to enhance the L1-mediated signal for apoptosis. Our results showed that indeed RC-L1 enhances the radiation-induced apoptosis as measured by immunoblotting using anti-Bax antibody (figure 5, panel D).

## Discussion

Insertion of an L1 copy into the genome necessitates the creation and repair of broken DNA. After L1 integration, the DNA ends are sealed and filled in, forming the target site duplications that flank a typical L1 insertion. Reactivation of L1 retrotransposition may interfere with potential *symbiotic' effects of L1 sequences such as their contribution to the global and local organization of the genome and the provision of gene regulatory sequences. Increased L1 retrotransposition may instead have a deleterious effect on the cell. It is widely presumed that L1 integration is random, therefore, increasing its mobility will most likely have neutral or negative consequences for the host cell. Even simply upregulating the L1 endonuclease in the absence of successful integration could be toxic to the cell by promoting the formation of additional DSBs, fostering chromosomal rearrangements and translocations. Furthermore, following DNA damage, cells initiate a repair response, which depends upon the close coordination of cell cycle checkpoints and activated DNA repair [22]. If the repair does not occur in a timely fashion or if the damage is massive, cell death by mechanisms involving apoptosis can occur [23,24].

L1-encoded endonuclease creates staggered DNA breaks, which enables newly-transposed L1 copies to integrate into the genome [7]. The outcome of single-strand breaks introduced by the endonuclease in a cell depends on several factors. A first factor is the cell cycle phase. Nicks in S-phase are most problematic, because they can be converted into double-strand breaks by the replication complex. A second factor is the DNA repair competency and capacity of the cell which may differ between normal and cancer cells. Thirdly, the presence of L1 RNA and other proteins at the nicked site may influence the type and efficiency of repair.

In a recent study, Goodier et al., have mapped a functional nucleolar localization signal in L1 ORF2. They showed that L1 ORF1 is localized in the cytoplasm with a speckled pattern and colocalized with ORF2 in nucleoli in a subset of cells [9]. However, although wild-type ORF2 expression was repeatedly observed, detectable levels remained prohibitively low. One cause of poor detection could be cell toxicity induced by nicking of genomic DNA by the endonuclease [9]. Similarly, early events in retroviral replication include entry of the viral capsid with the accompanying enzymes reverse transcriptase and integrase (IN) followed by synthesis of a DNA copy of the viral RNA genome to form a preintegration complex. This complex then enters the nucleus, and integration is first detected at approximately 3–4 h postinfection [25]. Retroviral integration is catalyzed by integrase acting on specific sequences at the ends of the viral DNA and via a concerted cleavage-ligation reaction that is mechanistically similar to that catalyzed by RAG proteins during V(D)J recombination [26,27]. As a consequence of integrase-mediated joining, the host cell DNA suffers a DSB, but the ends are held together by single strand links to viral DNA. Postintegration repair of this intermediate is essential for the maintenance of host DNA integrity as well as the stable association of retroviral DNA with host chromosomes. Numerous lines of evidence [28-30] indicate that retroviral DNA elicits a DNA damage response and that the integration intermediate is repaired primarily viacomponents of the non-homologous end-joining (NHEJ) pathway. It is noteworthy that Daniel et al., [31] provided direct confirmation that cultured cells respond to retroviral DNA integration in the same way that they respond to DSBs produced by a variety of genotoxic agents or normal programmed events, namely, by massive phosphorylation of histone H2AX in the vicinity of the damage site. The second finding is that H2AX appears to be dispensable for postintegration repair. These observations lend independent support to a model in which the anchoring of broken DNA ends to facilitate their repair is a critical function of γ-H2AX [31]. Severe DNA damage can result in cell cycle arrest and apoptosis [32]. Both cell cycle arrest and apoptosis have been seen to accompany retrotransposition in severely stressed cells [33].

## Conclusion

This is the first demonstration that human L1 retrotransposition induces DNA damage, as indicated by γ-H2AX accumulation. It also reveals a correlation between L1 expression/retrotransposition and induction of apoptosis. Taken together, the data imply that DNA nicks created by RC-L1 expression and retrotransposition are sensed as a DNA damaging event, which leads to apoptosis in cancer cells. Obviously further studies are needed to test whether additional components in the DNA damage recognition response, in particular NMR complex (Nbs1, MRE11, Rad50) and/or ATM are involved in signaling RC-L1 retrotransposition effects on these cells. In addition, while we realize that it is most likely the impact of the "active" form of L1, or RC-L1 that induces this DNA damage response, it will be of interest to clarify whether it is because of DNA double strand breaks or other intermediates in the retrotransposition cycle. While this manuscript was under review two other independent reports have been published by two different groups leading to the same conclusion indicating the activation of γ-H2AX in response to L1 expression and retrotransposition through the creation of DNA double strand breaks [34,35]

## Methods

### Cells, plasmids, and antibodies

MCF-7 and T47D human carcinoma cells were grown in DMEM (Gibco-BRL) supplemented with 10% fetal calf serum and 1% penicillin-streptomycin. L1-EGFP construct was obtained from E. Luning Prak (University of Pennsylvania, PA, USA). The anti-L1 ORF1 rabbit polyclonal antibody was a gift from Gerald Schumann (Paul-Ehrlich-Institut, Langen, Germany). The anti-L1-ORF2 rabbit polyclonal antibody was a gift from John Goodier (University of Pennsylvania, PA, USA). Anti-α-tubulin mouse monoclonal antibody from Sigma. Anti-γ-H2AX rabbit polyclonal antibody from Cell Signaling Technologies and FITC-conjugated secondary antibody from ICN Biomedicals, Inc.

### Cell transfection and selection

Cells were seeded in 6-well plates with about 4 × 10^5 ^cells/well and grown to 70% confluency in DMEM complete medium. Cells were transfected with the L1-EGFP construct using Lipofectamin 2000 transfection reagent (Invitrogen) following the manufacturer's protocol. Each transfection well received 2 μg plasmid DNA, 6 μl transfection reagent and 2 ml DMEM complete medium. Antibiotic selection was begun 24 h after transfection. Puromycin-resistant cells (pur^R^) were selected by growth in DMEM complete medium containing 10 μg/ml puromycin.

### Isolation of genomic DNA and PCR analysis

Genomic DNA was isolated using Qiagen Blood & Cell Culture DNA Mini Kit Kit following the manufacturer's protocol. The oligonucleotides used for PCR were GFP968F (5' GCACCATCTTCTTCAAGGACGAC-3') and GFP1013R (5'-TCTTTGCTCAGGGCGGACTG-3'). Amplifications were performed in 50 μl containing 1.25 U Ampli*Taq *Gold polymerase (Roche), 2.5 mM MgCl_2_, 1 × GeneAmp PCR Gold buffer (Roche), 0.2 mM each dNTP, 200 ng of each oligonucleotide primer and ~500 ng genomic DNA or 70 ng plasmid DNA template. After an initial step at 94°C (15 min), 35 cycles of amplification were performed (30 s at 94°C, 30 s at 59°C, 2 min at 72°C), followed by a final step at 72°C (10 min).

### Western blots

This procedure was performed as previously described [14]. Primary antibodies used were: anti-L1 ORF1 rabbit polyclonal (1:500 dilution), anti-L1 ORF2 rabbit polyclonal (1:200 dilution), anti-γ-H2AX rabbit polyclonal (1:1000 dilution), or anti-γ-tubulin mouse monoclonal (1:1000). HRP-conjugated secondary antibodies (Bio-Rad) were used. The detection was performed using the Immun-Star HRP Detection kit (Bio-Rad).

### Immunofluorescence

This procedure was performed as described in Haoudi et al [15]. Primary antibodies used were: anti-L1 ORF1 rabbit polyclonal (1:50 dilution), anti-L1 ORF2 rabbit polyclonal (1:100 dilution), anti-γ-H2AX rabbit polyclonal (1:500 dilution). The secondary antibody Alex-Fluor anti-rabbit (Molecular Probes) was used. The cells were viewed using a Zeiss LSM510 confocal microscope outfitted with Metamorph software. For quantitative analysis, foci were counted by eye during the imaging process using a 63 objective. In a single experiment, cell counting was performed until at least 40 cells and 40 foci were registered/sample. For data points that were derived from a single experiment, the error bars represent the SE from the analysis of the number of cells analyzed. For data points that were derived from more than one experiment, the error bars represent either the SE from the number of cells analyzed in the single experiments or the SE between the different experiments (whichever is highest).

### Flow cytometry

This procedure was performed as previously described [15]. To analyze the cell cycle profile, MCF-7 cells were either mock transfected or transfected with GFP or RC-L1. Forty eight hours later, RC-L1 transfected cells were subjected to puromycin selection, then DNA flow analysis was conducted on a BD Biosciences FACScan and analyzed with MODFIT software.

### Caspase 3 assay

Caspase 3 assay was performed using BD ApoAlert Caspase colorimetric assay kit following the manufacturer's recommendations.

### Gamma irradiation

T47D breast cancer cells were transfected with L1 plasmid as described above. Cells were then irradiated with 5 Gy using a ^137^Cesium source. Cells were returned to culture then selected for puromycin resistance. Whole cell lysates were then collected and subjected to immunoblotting using anti-Bax antibody, as described above.

## Abbreviations

LINE-1, long interspersed nuclear element 1; ERVs, endogenous retroviruses; LTRs, long terminal repeats; RC-L1, retrotransposition-competent L1; ORF, open reading frame; TPRT, target-primed reverse transcription; RNP, ribonucleoprotein particle ; PCR, polymerase chain reaction; DMEM, Dulbecco's modified Eagle's Medium; PBS, phosphate buffered saline; FITC, fluorescein isothiocyanate; FACS, fluorescence-activated cell sorting; EGFP, Enhanced green fluorescence protein; pur^R^, puromycin resistant; Mgcl_2_, magnesium chloride; dNTP, deoxyribonucleotide triphosphate; HRP, Horseradish peroxidase; bp, base pair; DSBs, double strand breaks; NHEJ, non-homologous end-joining.

## Competing interests

The author(s) declare that they have no competing interests.

## Authors' contributions

SMB carried out most of the experiments. RGG participated in the manuscript drafting and its critical reading. OJS participated in experimental design and discussion as well as in critical manuscript revision. AH designed the experimental approaches, contributed to cell culture, γ-H2AX foci formation assay and contributed to manuscript drafting. All authors approved the final manuscript.
